# Prioritization of Appearance over Health and Temperament Is Detrimental to the Welfare of Purebred Dogs and Cats

**DOI:** 10.3390/ani14071003

**Published:** 2024-03-25

**Authors:** Elodie Morel, Laureline Malineau, Charlotte Venet, Virginie Gaillard, Franck Péron

**Affiliations:** Royal Canin, 30470 Aimargues, France; elodie.morel@royalcanin.com (E.M.); laureline.malineau@royalcanin.com (L.M.); charlotte.venet1@royalcanin.com (C.V.); franck.peron@royalcanin.com (F.P.)

**Keywords:** brachycephaly, breed, breed-related diseases, BOAS, conformation, extreme, fashion, health, sustainable breeding, welfare

## Abstract

**Simple Summary:**

The welfare of purebred dogs and cats is being undermined by fashion trends that popularize extreme shapes. Official descriptions of breeds require their distinctive characteristics to be compatible with good health. Nevertheless, extremes within breeds that compromise health, referred to as hypertypes, have become trendy. For example, when flat faces are taken to the extreme, dogs and cats may be unable to breathe normally, which affects all aspects of their lives. Individuals such as breeders, owners and veterinarians, and organizations in the whole community surrounding purebred dogs and cats must collaborate to prevent the breeding of hypertypes. Fashion-fueled breeding of hypertypes, whether or not illegal, must also be tackled because its negative effects can extend beyond what is visible to unsuspecting owners choosing their puppy or kitten. The problem is complex, and the solution involves many bodies working together. However, the message is simple: health, temperament, and well-being must be prioritized over appearance. Putting this into practice will help protect the much-valued diversity of breeds.

**Abstract:**

Fashions in the appearance of purebred dogs and cats are encouraged by celebrity culture, social media, and online impulse buying. The popularity of characteristics perceived as cute, quirky, and anthropomorphic has driven increasingly exaggerated breed features appealing to aesthetics rather than health. ‘Hypertypes’ of some breeds have emerged that take a breed’s distinctive appearance to extremes beyond the intended interpretation of breed standards. This has severe, direct and indirect health and welfare consequences. Extreme conformations are associated with chronic health conditions including brachycephalic obstructive airway disorder, ocular, dental, skin, and musculoskeletal disorders. Puppy and kitten farms and illegal traders that meet the demand for hypertypes are associated with poor husbandry that neglects the physical, behavioral, and mental health of parents and offspring. A multidimensional approach involving collaboration between breeders, geneticists, owners, veterinarians, kennel clubs, cat fanciers’ associations, animal charities, the academic and research communities, commercial enterprises, and governments is needed to safeguard breeds and tackle these challenges. There are many ongoing initiatives by national kennel clubs and global partnerships to educate pet owners and support responsible pet ownership and sustainable breeding. The resounding message is that health, temperament, and well-being must be prioritized over appearance.

## 1. Introduction

Selective breeding of dogs by humans to fulfill specific functions commenced thousands of years ago, and one of the first known descriptions of breed standards is found in Cynegeticus by the Greek philosopher Xenophon (430–355 BC) [1]. Initially, breeding selected traits that would assist man in activities sustaining daily life, including hunting, transport, herding, and guarding, and also providing companionship was prioritized. Although cats would help with vermin control, the origin of breeds is probably attributable to aesthetically pleasing coat colors and markings to a greater extent than for dogs [2]. Fast forward to the Victorian era, and we see a major shift in focus towards selective breeding for appearance, the start of dog and cat shows, the foundation of kennel clubs and cat fancy associations, and systematic breed standardization [3]. Today, a large number of dog breeds are recognized by kennel clubs around the world. For example, the Fédération Cynologique Internationale (FCI) recognizes 356 breeds of dog, the Royal Kennel Club (RKC) 222, the American Kennel Club (AKA) 200, and the Canadian Kennel Club (CKC) 187. There are fewer cat breeds; the Cat Fanciers’ Association (CFA) recognizes 45 breeds, the Fédération Internationale Féline 48, and the International Cat Association (TICA) 73.

Dog and cat breeds reflect humankind’s history and culture, past and present. The wide variety of breeds help cement special relationships with humans having diverse lifestyles and preferences. There is merit in both the preservation of historic breeds and improvements in modern breed characteristics to ensure that relationships continue to be mutually beneficial. Dog and cat breeds can be a ‘friend’ or ‘foe’ to dog and cat welfare. Standard breed characteristics allow prospective owners to select breeds that best match their lifestyle, environment, expectations, and personality, thereby promoting the welfare of the pet and a healthy, happy long-lasting relationship. However, the physical appearance of breeds has increasingly become part of a consumer-driven fashion trade in which there is little consideration for health and welfare. This is not a modern phenomenon; pets were part of the conspicuous consumption of Victorians and were bred increasingly for their fashion value. The fast-paced culture of modern society and rapid communications means that cat and dog breeds are subject to the vagaries of fashion on a scale and way unlike that previously. Selection for extreme conformations has produced highly exaggerated features in some breeds, known as hypertypes. The welfare implications of these are of increasing concern to society [4,5,6,7].

Selective breeding of dogs and cats for extremes of appearance that cause ill health has been described as the “dark side of beauty” and “genetic abuse” [8]. We believe that this is unethical, but it does not make selective breeding of healthy dogs and cats with distinctive appearances unethical [9].

In this article, we will consider how genetic selection has produced breed dispositions to diseases, highlight detrimental trends for hypertypes in some dog and cat breeds, and unravel drivers of breed hypertypes. We will propose a multidimensional approach to promote breeding for health, temperament, and well-being rather than fashion trends; collaboration between all stakeholders concerned with pet welfare is needed.

## 2. The Genetic Realities of Heritable Disorders in Dog and Cat Breeds

Many gene variants in dogs and cats are associated with disease regardless of the breed background on which they occur [10]. ‘Ancient’ disease-associated alleles are likely to result in inherited disease in multiple breeds and they are also found in mixed-breed dogs [7]. However, a key difference between purebred and mixed-breed dogs with respect to monogenic (single gene) disorders is that the former are in general more likely to be homozygous for recessive disease variants and therefore more likely to develop the disease [11]. This occurs because of the inbreeding inherent in the development of breeds, which, combined with small founder populations and population bottle necks, has reduced genetic diversity. Mixed-breed dogs are not immune to homozygous disease-associated alleles; it depends upon the breed mix because, as mentioned, some deleterious alleles are associated with more than one breed.

Strong selection for a desired trait can coincide with unintended selection for a disease-related allelic variant, which may be physiologically unrelated to the selected trait. For example, selection for distinctive black spots in Dalmatians has resulted in homozygosity for a recessive mutation of the *SLC2A9* gene, which causes hyperuricosuria and thereby a risk of urate urolithiasis [12]. As another example, Persian cats have been highly selected for their brachycephalic conformation, but this anatomy is not related to the pathogenesis of heritable autosomal dominant polycystic kidney disease that has been inadvertently enriched in the breed [13,14]. The phenomenon of gene linkage is one of the ways that desirable traits become inherited simultaneously with undesirable traits. If a desired allelic variant is in close proximity in the chromosome to another gene with a disease-associated allele, positive selection for one can simultaneously and unintentionally select for the other.

Knowledge of the history of more recent breeds can explain why a disease predisposition can occur in two breeds with some striking differences in appearance. The Devon Rex and Sphynx breeds have very different coats, the first having curly hair and the latter being nearly hairless. However, both breeds are associated with an autosomal recessive neuromuscular condition. In fact, the two breeds are closely related because during the development of the Sphynx breed, there was outcross breeding with the Devon Rex to increase the gene pool. The deleterious mutation responsible for congenital myasthenic syndrome was present in the Devon Rex breed when it was first established and spread to the Sphynx via well-intentioned outcrossing [15].

Understanding the nature of genetic predispositions apparently associated with a breed characteristic is much more complicated when the disease is influenced by multiple genes (polygenic). For example, the risk of osteosarcoma is highest in dogs selected for long legs/large body mass, such as the Great Dane and Rottweiler, but both size and cancer risk are polygenic traits, so there will not be a simple genetic relationship [16].

An inherited disease predisposition can also arise because a deleterious allelic variant is directly responsible for a morphological or other aesthetically desirable trait in a breed. For example, Rhodesian Ridge Back dogs are characterized by their prominent hair ridge, which is a consequence of mutations in three genes for fibroblast growth factors that also predispose to the congenital dermoid sinus [17,18]. Extreme-white coat patterning that has been selected in several breeds including the Dalmatian is associated with a predisposition to congenital sensorineural deafness [19]. At least one genetic link between the two is believed to be the gene for melanocyte-inducing transcription factor (*MITF*) [20,21]. This is an important controller of melanocyte development, which affects pigmentation and also hearing, because melanocytes in the inner ear are essential for its proper functioning.

Some extreme morphological characteristics in purebred dogs and cats can predispose the animal to disease syndromes directly related to the desired and selected anatomy. The relationship between the physical appearance of hypertypes and disease pathogenesis is discussed in Section 3.

Breed predisposition for a disease does not necessarily equate to a high incidence of the disease. The rapidly expanding knowledge base of geneticists combined with the professionalism of responsible breeders contributes to the development of breeding plans that prioritize health as well as preserving and improving breed characteristics. We will return to responsible breeding as a force for good in Section 6.

## 3. Hypertypes and Their Health Consequences in Some Fashionable Dog and Cat Breeds

Over time, the appearance of many breeds has changed significantly, with the exaggeration of features in some breeds leading to hypertypes. We define a hypertype as a phenotype that shows extreme or exaggerated breed characteristics that extend beyond the breed standard and that are potentially detrimental to the health and well-being of the animal [22]. A hypertype may result from a tendentious interpretation of the breed standard; a balanced interpretation of breed standards should not result in hypertypes [23]. Trends towards hypertypes reflect society’s relentless pursuit of fashion, documented as extreme fluctuations (boom and bust) in breed popularity [24]. Recent breed preferences indicate the importance of human attraction to physical traits perceived as cute, anthropomorphic, paedomorphic or quirky, and size extremes (miniaturization and gigantism). Examples include wide-set eyes, flat faces, domed heads, and excessively wrinkled skin. There may be the perception that ‘more’ of a breed feature is better. The extremes defining hypertypes are not only often directly responsible for clinical disease and pain, but they can be linked to behavioral and welfare problems. For example, characteristics that impede locomotion or breathing are likely to restrict the animal’s ability to exercise and exhibit natural play behavior. Social signaling may be more difficult for animals with exaggerated brachycephaly that inhibits facial expressions or with tightly curly tails that are relatively immobile [25].

Examples of hypertypes of some popular breeds are described below.

### 3.1. Dogs

**Figure 1:**
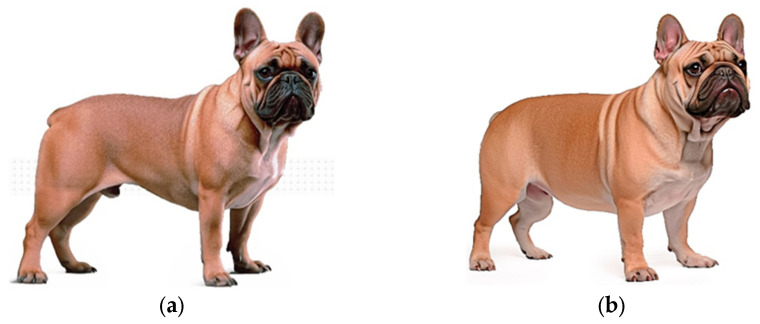
(**a**) French Bulldog conforming to the breed standard; (**b**) hypertype of French Bulldog. Illustrations reproduced with the permission of Royal Canin^®^. (Supplementary Materials).

Brachycephalic hypertypes have come into sharp focus with the rising popularity of Bulldogs, French Bulldogs and Pugs. Exaggerated external features result from aberrant skeletal and soft tissue anatomy that cause health and well-being issues. Extreme brachycephaly in dogs is manifested by a severely shortened muzzle, a rounded and often massive head, stenotic nares, a pronounced underbite, reduced frontal sinuses, oversized nasal turbinates, and a relative excess of upper respiratory tract soft tissue, e.g., elongated soft palate that impedes airflow (Figure 1) [26]. These abnormalities are responsible for brachycephalic obstructive airway syndrome (BOAS), which manifests as labored, noisy breathing, chronic shortness of breath, exercise, and heat intolerance; low oxygen levels can cause collapse and sometimes death [26]. Brachycephalic hypertypes are also susceptible to ocular surface disorders collectively called brachycephalic ocular syndrome (BOS) [27]. Shallow orbits resulting in exophthalmia and excessively large palpebral fissures, and abnormalities in eyelids, blinking, and the tear film increase the risk of dry eye disease and ulcerative keratitis. Other health issues associated with brachycephalic hypertypes include dental problems due to severe crowding of teeth, malocclusion, and rotation of premolars, and dystocia due to disproportionally large heads.

Changes in the head shape of Cavalier King Charles Spaniels have also occurred since the early 20th century and a more pronounced brachycephalic phenotype with a domed head and deep stop have become popular despite the breed standard [28]. The Cavalier King Charles Spaniel breed has a high prevalence of a complex and painful developmental abnormality of the skull and craniocervical vertebrae called Chiari-like malformation [29]. There is overcrowding of the brain and cervical cord, and obstruction to the flow of cerebrospinal fluid predisposes the breed to syringomyelia, a neurological condition with clinical sings including scoliosis, muscle atrophy and weakness [29]. Importantly, the exaggerated brachycephaly and domed head of the popular hypertype of this breed are risk factors for syringomyelia [28].

The tightly curled corkscrew tail in Bulldogs, French Bulldogs, Pugs and Boston Terriers is caused by congenital malformations of the vertebral bodies [30]. When severe, tail malformations are positively associated with malformations in the thoracolumbar vertebrae, these are in turn linked to neurological deficits. It has been suggested that clinical assessment of the degree of tail malformation may be an easy and accurate way to inform breeding suitability [30].

A hypertype of the Dachshund breed has become increasingly popular in which the characteristically long and low phenotype is so extreme that it impedes free movement and increases both the risk of thoracolumbar intervertebral disc extrusion and the severity of clinical signs when this occurs [31].

Hypertypes of several dog breeds including the French Bulldog, Bulldog and Shar pei have excessive skin folds that predispose them to recurrent and chronic dermatitis and pyoderma [32,33,34]. Although it is possible to treat these conditions they are nevertheless painful and many may not be addressed because owners frequently do not recognize the clinical signs (37% of owners of English Bulldogs in a Finnish survey [32,35]). Corrective skin surgery may be indicated in severe cases, but the stress and pain of the procedure also has welfare implications.

**Figure 2:**
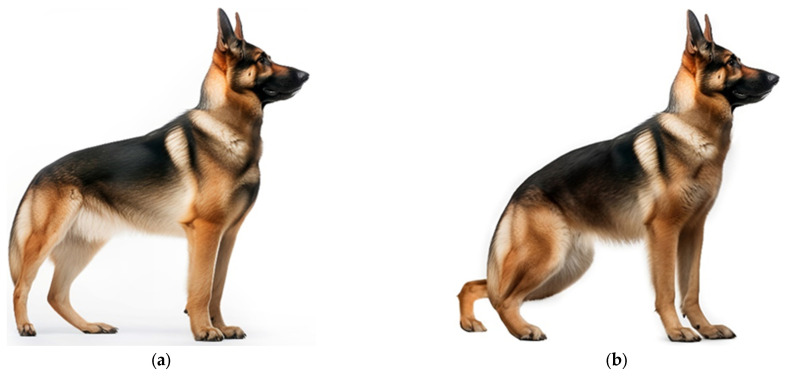
(**a**) German Shepherd conforming to the breed standard; (**b**) hypertype of German Shepherd. Illustrations reproduced with the permission of Royal Canin^®^. (Supplementary Materials).

Profound changes in the conformation of the German Shepherd dog have resulted in an exaggerated sloping back from the withers to the base of the tail, and acute angulation of the hind limbs (Figure 2). These characteristics adversely impact standing posture and gait and reduce the power of the hind limbs [36]. In a UK study, the two most common causes of death in German Shepherd dogs were musculoskeletal disorders and inability to stand, although the study did not investigate causality [37].

### 3.2. Cats

**Figure 3:**
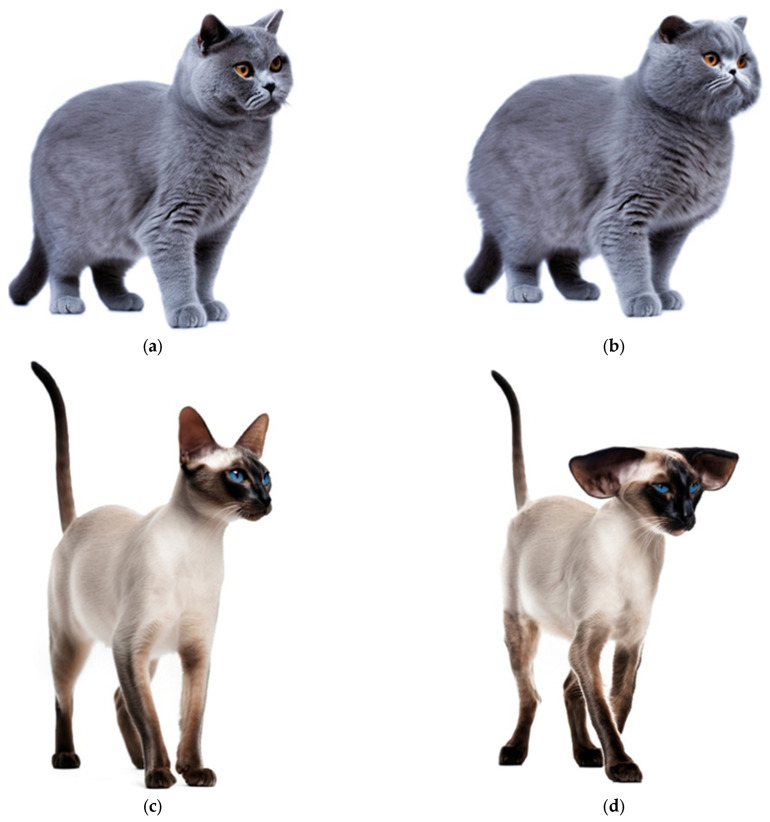
(**a**) British Shorthair conforming to the breed standard; (**b**) hypertype of British Shorthair; (**c**) Siamese conforming to the breed standard; (**d**) hypertype of Siamese. Illustrations reproduced with the permission of Royal Canin^®^. (Supplementary Materials).

There is quantitatively less research into hypertypes of cat breeds compared with dogs. Nevertheless, negative health implications of extreme brachycephaly in popular breeds such as the Persian, Exotic Shorthair, and British Shorthair are documented and supported by computed tomography (Figure 3) [38,39,40]. The characteristics of the traditional doll-face Persian cat have given way to the exaggerated brachycephalic peke-face Persian (Figure 3). Brachycephaly in hypertypes is associated with severe skull and brain abnormalities such as hydrocephalus [39]. The greater the brachycephaly the more likely the cat will have respiratory difficulties [38]. In primary practice in the UK, haircoat disorders were one of the most prevalent disorders in Persian cats [14]. It is hypothesized that brachycephaly, compounded by long coat hair, makes grooming inherently more difficult for this breed, thereby impacting basic selfcare and compromising welfare [14,41].

The more pronounced the brachycephaly of Persian and Exotic Shorthair cats is, the greater the dorsal displacement of the facial bones, upper jaw, and canine teeth, which can be almost horizontal [40,42]. This anatomy displaces the nasolacrimal duct drainage system, impairing the drainage of tears and causing epiphora and chronic tear staining [40]. Oral and dental abnormalities in extreme brachycephalic cats are likely responsible for their predisposition to tooth resorption and periodontal disease [43]. More severe brachycephaly correlates positively with the degree of exophthalmos and stenotic nares [42].

Extreme elongated wedge-shaped heads and large ears in doliocephalic breeds such as Oriental and Siamese cats (Figure 3) may also be detrimental to health and behavior, although the evidence appears to be more limited [44,45,46].

The Maine Coon breed is popular in part for its large frame, which has progressively increased in recent years combined with a greater angulation of the hind limb and the development of a characteristic Maine Coon gait [47]. The breed has a relatively high incidence of hip dysplasia [48,49,50,51] that is more severe than in other breeds with the same degree of laxity in the hip joint (Norberg angle) [47]. Severity of hip dysplasia is genetically correlated with body mass and when breeders select for body types larger than average, they may also be selecting for a higher risk of hip dysplasia [51].

## 4. Behavioral Hypertypes

The focus of this article is on hypertypes selected for physical appearance, but behavioral hypertypes also exist with potentially negative effects for well-being [52]. Behavioral characteristics are heritable [53], described in breed standards, and they can be evaluated by research tools such as the Canine Behavior and Research Questionnaire (C-BARQ) [54] and simple owner-targeted questionnaires [55]. Selection within a breed can be biased towards either show or working dogs. Genetic selection signatures have been found for example in German Shepherd dogs for chasing and aggression when comparing working lines with predominantly pet dogs [56]. Breeding for working versus show dogs has also selected for impulsivity trails [57]. These differences can become problematic when dogs with extremes of ‘working-related’ breed behaviors are bought as pets for home environments incompatible with these characteristics. The well-being of an animal inherently unable to adapt to the environment in which it is placed may be compromised by both their own stress and that of their owner. Anecdotally there is a fashion amongst pet owners of some breeds to seek puppies from working lines, without considering the potential for extremes of working-related behavior they would find difficult to manage.

## 5. Fashion Trends and the Welfare of Purebred Dogs and Cats: A Complex Picture

The popularity of dog breeds appears to be determined largely by fashion rather than breed health, longevity, and behavior [58,59]. Popular dog breeds are not inherently associated with poor health and well-being; the problems are associated with the hypertypes of these breeds and irresponsible breeding. Fashion is not restricted to breed type; chubby puppies and kittens in general have a cuteness appeal to some pet owners. This potentially encourages breeders to strive for higher rates of weight gain, or faster catch-up growth in low birthweight individuals. High growth rates are a risk factor for severe musculoskeletal disorders such as osteochondrosis in large and giant breed dogs, which causes pain and functional impairment, and may lead to euthanasia [60,61,62]. Fast growth rates are also associated with overweight in adulthood, and overweight and obesity are risk factors for many, diverse health disorders, e.g., diabetes, neoplasia, lower urinary tract disease [63]. When fashionable traits in appearance are prioritized over health, temperament, and well-being, the ramifications extend beyond any potential deleterious effects of the trait itself. The nature and extent of the issues depend upon the social and economic context surrounding acquisition and ownership of puppies and kittens. The two key aspects are choice of a particular ‘look’ and choice of breeder.

We live in a world of globally accessible visual media, targeted online advertising, and rapid communication networks including social media that sustain celebrity culture, viral videos and generally promote the power of outward appearances. The internet facilitates remote selection and purchase of puppies and kittens with the speed and convenience that people have come to expect for consumer goods and services. A survey in the UK found that nearly half of puppies advertised online were from just 10 breeds, and breeds renowned for hypertypes with conformational disorders were advertised more frequently than other breeds [64].

Recent fashion trends have been investigated through purchasing practices pre the COVID-19 pandemic (2019) and post the pandemic peak in the UK (2021). Owners in 2021 were less likely to seek a puppy or kitten considered generally healthy, less likely to look for a breeder that performed health tests, and less likely to use the RKC website for background information [65]. The behavior of prospective dog owners in the UK differs according to the breed type they choose [66]. Owners of brachycephalic breeds reported that appearance was the most influential factor on their breed choice; the perceived general health of the breed had less influence compared with owners of non-brachycephalic breeds [66]. How brachycephalic puppies were purchased was also less health-focused compared with non-brachycephalic puppies; owners were less likely to have asked to see the health records of the dam and sire, and these records were less likely to be available [66]. Similarly in Denmark owners of French Bulldogs were mainly interested in appearance and personality, while owners of Chihuahuas seemed to prioritize ease of finding and obtaining the dog [67].

There are also worrying trends in purchasing patterns for kittens. Owners of brachycephalic breeds report appearance (predominantly cuteness, funny looks and the human-like face) and behavioral traits as the main motivators for buying these breeds; most were unaware of breed-associated breathing disorders [68]. This might reflect the use of non-registered breeders and potentially a greater risk of extreme phenotypes. Additionally, when purchasing a kitten from a breeder, owners of brachycephalic breeds were less likely than owners of other purebred cats to know if the parents had undergone health tests and, when it was known, testing was less likely for both parents [69].

A high demand for particular breeds of puppies and kittens and a focus on outward appearance provides ideal circumstances for unscrupulous breeding. Many aspects of irresponsible breeding are not governed by legislation, and where laws do exist, effective enforcement may be lacking. Unlicensed or unregistered breeders do not necessarily have poor breeding standards, but they are less accountable to external authorities. Poor practices can occur in any size of breeding establishment, but the business model of puppy and kitten farms combined with internet advertising allows them to capitalize on consumer demand for immediate availability by undesirable means [64]. High puppy and kitten prices associated with supply pressure tempt illegal or unscrupulous pet traders who lack either the knowledge of, and/or motivation to follow best practice breeding and husbandry. They can undercut the prices of responsible breeders, and price conscious prospective owners may be ignorant of how low prices are achieved. The harm caused by suppliers of puppies and kittens with poor standards and a sole interest in financial gain is far reaching. Puppy and kitten farms fall into this category by definition. Differences between other large-scale commercial breeders and occasional or small-scale breeders exist, but breeding and husbandry practices in either can be well considered or detrimental [70]. Any breeder intentionally selecting for hypertypes to meet consumer demand and breeding without well-informed consideration of the genetics, medical history, and temperament of parents increases the risk of both conformational and non-conformational inherited disorders. Poor environmental conditions and treatment of animals in puppy farms are welfare issues for both parents and offspring per se, independent of breeding genetics. Failure to meet developmental needs and provide appropriate socialization, nutrition, and veterinary care, are just some of the concerns [71]. Altogether, irresponsible breeding and selling practices augment the problems of irresponsible buying.

The deleterious genetic consequences of irresponsible inbreeding extend beyond the individual and the trait being intentionally selected. Inbreeding reduces the genetic diversity of a breed and increases homozygosity, including homozygosity for deleterious alleles. The effective genetic population size can decrease even as the total number of dogs or cats of that breed overall increases [72]. Ultimately inbreeding is associated with increased morbidity, increased risk of inherited disease, reduced litter size, survival, and fertility [73,74,75,76,77]. Excessive inbreeding therefore risks the disappearance of some breeds over time.

## 6. A Multidimensional Approach to Prioritizing the Health, Temperament, and Well-Being of Dogs and Cats

A multidimensional approach is needed to build a healthy ecosystem of dog and cat breeding that rejects any tendency towards hypertypes and prioritizes health, temperament, and well-being over fashionable looks. All stakeholders in pet ownership have interrelated roles to play in such an ecosystem. Provision of education and practical tools and materials are core strategies across stakeholders and communication platforms. Innovation is important but many initiatives are already in existence and should be fully leveraged. Interventions and recommendations that relate directly to the pathophysiology of hypertypes should be made with the advice of geneticists. Without their expertise, breeding programs to eradicate extreme breed characteristics could reduce genetic diversity so severely that other heritable problems arise, which may even endanger breed survival. Policy decisions by regulatory and governmental bodies as well as breed societies, kennel clubs and cat fanciers’ associations must be informed by up-to-date genetic knowledge.

### 6.1. Breeders

Breeders have a special role as guardians of the health and temperament of breeds. Education of breeders on genetic inheritance of disorders empowers them to select dams and sires based on health, and to make the best use of genetic and phenotypic selection tools. Because there can be complex genetic associations of appearance and heritable disease, education on inbreeding, line breeding, and excessive use of a small number of sires should include the concepts of genetic linkage and polygenic inheritance. Breeders should also understand the importance of breeding towards low disorder risk as well as breeding away from high disorder risk [78]. Expert support from veterinarians and geneticists should be available to advise on breeding programs.

Breeders can screen potential parents for disease-associated alleles and genetically determined conformational traits with commercially available DNA tests. The use of technology to analyze single nucleotide polymorphisms (SNPs) rather than short tandem repeats (STRs) allows for more accurate identification of individual dogs and cats and their parentage [79] (e.g., MyDogDNA Select^®^). Some kennel clubs stipulate testing and reporting quality standards for genetic tests [80]. Breeders should also be aware of the limitations of genetic testing, especially with respect to polygenic disorders such as hip dysplasia. A balanced breeding program should consider genetic tests alongside other clinical diseases for which genetic tests are not available [81].

Pedigree registration of purebred dogs and cats gives breeders access to information on the health of individual animals and numerous tools to help them prioritize health in their breeding plans [82]. Depending upon the kennel club or cat fanciers’ association, these can include various health records, results of disease screening (e.g., radiographic scoring for hip and elbow dysplasia, deafness testing, eye screening), DNA testing, and calculators for coefficients of inbreeding. For example, the Société Central Canine (SCC [French Kennel Club]) has an internet portal for breeders that provides health information for potential dams and sires and allows them to generate a virtual pedigree. The RKC and Swedish Kennel Club are amongst those that provide Estimated Breeding Values for hip and elbow dysplasia based on screening of the individual and its relatives. This is especially helpful for polygenic disorders with complex inheritance patterns, which may be subclinical or take time to develop. The Governing Council of the Cat Fancy (GCCF) holds a genetic register of cats that are a potential breeding risk, either because they are a proven carrier of a heritable disease, or because they are untested for a deleterious genetic trait known to be present within the gene pool. This can be used in managed breeding programs to eliminate known genetic anomalies and prevent their spread to other breeds of cat when outcrosses are permitted.

Other tools and educational initiatives extend beyond assessment of the breeding suitability of individuals. For example, the Breed Health and Conservation Plan Project run by the RKC provides a complete evidence-based view of breeds to support balanced breeding decisions. The SCC provides training for the Attestation of Knowledge for Pets of Domestic Species, which is a legal requirement in France for breeders and sellers of cats and dogs. Royal Canin’s PROactive program is another source of support that was launched in 2019 and deployed globally in partnership with the FCI [83]. This encompasses resources for breeders such as personalized diagnostic, e-learning courses and practical guides, exclusive conferences, and other opportunities to share expertise and knowledge.

Recognition of breed hypertypes and their health implications must be high on the agenda of all breeders. Simple critical assessment of appearance and morphometric measurements can be used. Although surgical correction or medical treatment for extreme phenotypes may be available, this can often only partly improve the animal’s welfare, and the focus should be on breeding to minimize the need for interventions. Education is required to clarify that dogs and cats can only conform to breed standards if they are functionally and clinically healthy; hypertypes are phenotypes beyond the boundaries of breed standards [84]. Magnetic-resonance imaging to screen for syringomyelia could be made compulsory for pedigree registrations of all breeding stock of susceptible dog breeds [85]. Breeders should be encouraged to have Bulldogs, French Bulldogs and Pugs tested for BOAS using the RKC and University of Cambridge’s respiratory function grading scheme (RFGS) [86] or another similar functional test such as the brachycephalic exercise aptitude test for health (BREATHE) used by the SSC and the Ente Nazionale della Cinofilia Italiana (ENCI [the Italian Kennel Club]) [87]. Breeding programs must balance phenotype and genotype. For example, the exclusion of French Bulldogs with any degree of nasal stenosis from breeding would promote healthy dogs that still complied with the SCC’s breed standard. However, given the high prevalence of nasal stenosis, it would be advisable to stage such a program by initially avoiding breeding from dogs with severely stenotic nares, in order to prevent a precipitous reduction in the breed’s gene pool [88].

In addition to healthy breeding programs, breeders are responsible for giving puppies and kittens the best start in life. Optimal early nutrition, socialization and weaning may reduce the risk of dogs and cats developing chronic disorders in adulthood such as obesity and behavioral problems [63]. In large and giant dogs, correct nutrition is especially important to reduce the risk of musculoskeletal disorders. In other species, effects of early-life environmental factors on later health can be mediated by epigenetic modifications to DNA that ‘program’ the expression of genes [89]. Although the sequence of the DNA is not changed, some of these epigenetic changes appear to heritable [90,91]. If this type of inheritance is confirmed in dogs and cats, the significance of providing good environments for young puppies and kittens as well as their parents could extend beyond the individuals to future generations.

### 6.2. Kennel Clubs, Cat Fanciers’ Associations and Other Organizations Registering Purebred Dogs or Cats

The previous section has highlighted some of the many ways that kennel clubs and cat fanciers’ associations can and are supporting responsible breeders. There are large differences in how kennel clubs around the world are approaching breeding policies and management, but they are making great efforts to collect and provide information on breeding and health [6]. It is important that these initiatives are publicized and evolve and expand as science uncovers more genetic disease associations and more phenotypic tools are validated.

Keepers of breed standards such as the AKC, the RKC, the SSC, the GCFC, the Club Livre Officiel des Origines Félines (LOOF) and numerous other national pedigree associations around the world have special responsibilities. Even in the absence of changes in breed standards, detrimental changes in the characteristics of some breeds have occurred over time as mentioned above. Changes in breed standards may therefore be beneficial to explicitly exclude specific hypertypes. For example, wording was added to the RKC’s German Shepherd dog breeding standard in 2016 to emphasize the need for a comfortable standing posture and sound gait [92]. Objective assessments may be particularly helpful. The breeding standard for the Dachshund, as defined by the Verband für das Deutsche Hundewesen (German Kennel Club) specifies proportional dimensions for ground clearance, and the RKC introduced a variant of this in 2021 [93,94]. The Nordic Kennel Union advocates incorporating craniofacial ratio specifications in the breed standards of brachycephalic dogs [95].

National kennel clubs and cat fanciers’ associations and international registries to which they are affiliated have overarching positions discouraging hypertypes. For example, the FCI statutes make it clear that the health, temperament and behavior are of utmost importance in breed standards [96]. The FCI Committee for Dog Welfare and Health aims to spread scientific knowledge and put it into practice for the benefit of dogs worldwide, supporting the work of breeders, breed clubs and national canine clubs.

There will always be a subjective element to the interpretation of breed standards. The SCC emphases that current breed standards should not result in hypertypes; a balanced interpretation is needed by breeders, show judges and veterinarians. Show judges have a critical role in helping to set standards of health, well-being, temperament and function [97]. By selecting show winners they influence the selection of dams and sires and thereby the characteristics of future litters. Although national bodies are responsible for the training of show judges, the FCI also sets standards [97,98]. Exaggerated breed characteristics that can lead to health, behavior or movement problems, should never be awarded a qualification “Excellent” [98]. Judges can complete forms on breed-specific problems encountered during judging and these are used by relevant national association the national canine organization to enhance breed health [98]. The Nordic Kennel Union of five Nordic countries has Breed Specific Instructions (BSI) on exaggerations in pedigree dogs for show judges [99].

As well as documenting specific health and DNA test results for individuals, kennel clubs and cat fanciers’ associations can mandate them for breeding animals by requiring satisfactory test results from dams and sires before their litters can be given pedigree registrations. Another approach is voluntary quality assurance schemes to encourage breeders to maintain high welfare standards and sign-post prospective owners to breeders adhering to specific criteria of responsible breeding. For example, the RKC’s Assured Breeder Scheme requires breeding animals to be screened for certain health predispositions [100]. The AKC has a Bred With H.E.A.R.T Program in which breeders commit to health testing, AKC-provided or approved continuing breeder education, and husbandry standards [101]. Members of the Breeder Scheme of the GCCF are required to select all breeding cats for health and temperament [102]. The reliability of test results is essential. For example, hip dysplasia scoring is ideally performed by a veterinarian with radiography qualifications, because the score depends upon the quality and skilled interpretation of a radiograph. The use of a laxity index or distraction index is also recommended to complement the hip dysplasia classification used by the FCI [103].

### 6.3. Dog and Cat Owners

Education of dog and cat owners on careful selection of both the animal and breeder has never been more important to encourage cultural shift towards making health and welfare fashionable. The same communication channels that encourage irresponsible buying can be used to promulgate best practice. Having a purebred pet, whether or not it has a registered pedigree, is no guarantee of health, and it is imperative that all pet owners understand the issues surrounding hypertypes. Epidemiological information on owners and their purchasing preferences can be used to target communications, both by content and channel. Messages that reach the general public are also valuable because society needs to be invested in animal welfare.

Messages about the welfare implications of hypertypes are needed to help owners understand that the exaggerated characteristics they perceive as cute and normal for the breed are in fact serious health and welfare issues, e.g., the WSAVA’s YouTube video on BOAS [104] and the position statement of the International Collaborative on Extreme Conformations in Dogs (ICECDogs) [105]. In one survey, more than half owners of dogs with confirmed BOAS did not perceive their pet to have a breathing problem [106].

There is plenty of authoritative and independent buying advice for prospective dog and cat owners from kennel clubs, cat fancier associations and charitable organizations. These set out the practical ‘how to’s’ that buyers need to choose both breeder and puppy or kitten in order to prioritize health, temperament, good veterinary care, welfare-conscious environments and husbandry, and good socialization for healthy behaviors. The Pup4Life checklist is one example of a user-friendly guide to choosing the right breeder for prospective owners [107]. Recommendations include buying in person to directly observe the premises and puppy or kitten on more than one occasion, checking the mother’s health and temperament, insisting on all the correct documentation and health information such as vaccination status and deworming. Buyers should expect a responsible breeder to ask questions about their suitability of their lifestyle for the breed in question. Impulse purchases should be strongly discouraged [108]. Campaigns such as those of Four Paws International provide powerful case studies to demonstrate the consequences for both owners and their pets when they are exploited by unscrupulous suppliers [109].

### 6.4. Small Animal Veterinarians and Veterinary Specialist Boards

Veterinary expertise is necessary to inform all activities in a multidimensional strategic approach to the healthy breeding of purebred pets. The role of individual veterinarians includes providing expert and persuasive advice, using, and encouraging the use of health-related tools such as genetic tests, standard growth curves, and proactive identification of breed-associated diseases, especially in hypertypes. Veterinary clinicians are well placed to educate owners and breeders on obesity and overweight which exacerbate BOAS and osteo-articular disorders. Growth should be monitored closely against standard growth curves to reduce the risk of overweight and musculoskeletal disease [110,111,112,113].

Various veterinary associations have position statements on responsible breeding. The Federation of European Companion Animal Veterinary Associations (FECAVA) and the Federation of Veterinarians of Europe (FVE) adopted the position that “health and welfare should go before looks” [114]. The British Small Animal Veterinary Society (BSAVA) strongly recommends that animals with extreme conformations that are detrimental to their health and welfare should not be used for breeding [115]. The position paper of the World Small Animal Veterinary Association’s (WSAVA’s) Hereditary Disease Committee calls on veterinarians and breeders to exclude animals with extreme conformations (e.g., extremes of size, skin folds, angulation and brachycephaly) predisposing to hereditary disease from breeding [116]. The American Veterinary Medical Association (AVMA) encourages veterinarians to undertake continuing education in genetic diseases of companion animals and to educate breeders, pet owners and the public on responsible breeding and pet ownership [117]. We recommend that the relevant European and American veterinary colleges of specialists recognized by AVMA or the European Board of Veterinary Specialization (EBVS) take a role with their own statements on breed hypertypes.

### 6.5. Non-Government Organizations, Welfare Charites, Researchers, Academics and Commercial Stakeholders

There is a wide range of non-government organizations, welfare charities, researchers, academics, and commercial stakeholders that share the same vision of health before appearance for purebred dogs and cats, promoting responsible pet ownership and responsible breeding. We encourage collaborative partnerships that maximize the different reach and capabilities of these stakeholders. These include access to breeders and owners for epidemiological research, educational materials, and events. The International Partnership for Dogs (IPFD) is one non-profit organization leading global, multistakeholder efforts to improve the health of purebred dogs. For example, it collaborates with geneticists and testing laboratories to promote rigorous and harmonized standards for genetic testing [118], provide unbiased information on test selection, and explain the meaning of the results. Diverse commercial interests in the ecosystem of purebred dogs and cats cannot be overlooked and can leverage quite different types of influence. For example, pet insurance companies can work with kennel clubs and cat fanciers’ associations to provide a financial incentive for owners to buy only individuals with certified pedigree health credentials. The lobbying of legislative bodies is another example where collective action is needed to generate change. Non-government organizations can apply pressure from the public and welfare-conscious breeders, welfare charities have direct experience of the consequences of irresponsible breeding and purchasing, and researchers have compelling evidence from genetic, epidemiological, and clinical studies. These stakeholders should work together with veterinary bodies and breed associations that can provide different insights into the issues and practical solutions.

### 6.6. Governments and Regulatory Bodies

As mentioned, unscrupulous or illegal trade in puppies and kittens is one consequence of a consumer led market for hypertypes. An increasingly close relationship between organized crime and dog dealing in hypertypes has been exposed in one documentary [119]. There is no single legislative solution to tackle low-welfare, high volume supply of puppies and kittens. For example, the UK government has prohibited commercial third-party sales of these pets [120], but this must work alongside stringent legislation on importing and exporting and other welfare regulation. In December 2023 the European Union published a proposal for legislation that would improve the traceability of dogs and cats across countries [121]. This would mandate electronic identification and registration of dogs and cats before being placed on the market or supplied, including when offered for sale or adoption online.

We believe that laws to govern the breeding, trade and ownership of dogs and cats will only be able to protect their health and safeguard the rich diversity of breeds if they are reasonable, enforceable, and non-discriminatory. Banning breeds could augment trading through illegal routes. It would fail to recognize the passion of many dog and cat owners for maintaining the diversity of current breeds. Banning ownership of specific breeds would risk stigmatizing owners and increasing the number of relinquishments. The fundamental challenge is hypertypes within breeds. Governments must work collaboratively with the many stakeholders above to devise and deploy targeted strategies. Supported by an appropriate and empowering legislative framework, these are the stakeholders that can make the changes needed. Within this framework we strongly advocate official recognition of professional breeders in all countries.

Regulations on advertising standards need to address advertisements for the sale of dog and cats hypertypes, and the use of images of hypertypes in the promotion of any products or other commercial communications to the public. The International Collaborative on Extreme Conformations in Dogs (ICECDogs) will be publishing guidelines on the use of images of dogs with extreme conformation. Advertising and branding are backdrops to many activities in daily life and can easily normalize or nurture fashions for hypertypes. Advertising regulations have a role to play, but education of companies, marketing agencies and media channels is needed for them to actively participate in positive change.

## 7. Conclusions

Undoubtedly dog and cat hypertypes of any parentage are welfare concerns. We believe however that actions targeting purebred hypertypes are likely to have the greatest impact because of the scope for control and regulation, and the overall mission of breeders of purebred dogs and cats. Fashion can directly and indirectly amplify welfare problems by driving increasingly exaggerated breed characteristics, fueling irresponsible breeding practices, and appealing to aesthetics rather than health, in addition to compromising genetic diversity and even threatening the future of the breed. The wonder of the many breeds of dogs and cats is special, and breeds can be distinctive without compromising welfare. “It is vital to breed for health whilst retaining the key features of the small animal breeds. A number of different strategies are already being implemented—moderation is the way forward” [Jane Ladlow MA VetMB CertVR CertSAS DipECVS MRCVS, Director of Clinical Research, University of Cambridge. Personal communication]. Key aspects of safeguarding breeds and making them sustainable are the professionalization of breeding, and responsible pet ownership. There are many other stakeholders with shared welfare goals, and collaboration in a multidimensional approach is needed to make the health, temperament, and well-being of dogs and cats paramount.

## Data Availability

Not applicable.

## Supplementary Materials

The following supporting information can be downloaded at: https://www.mdpi.com/article/10.3390/ani14071003/s1.
